# Surgical Site Infections due to Non-Tuberculous Mycobacteria

**Published:** 2018-06-30

**Authors:** Rohit Prasad Yadav, Bashudev Baskota, Rabin Ratna Ranjitkar, Sandesh Dahal

**Affiliations:** 1 Department of General Surgery, Nobel Medical College, Biratnagar, Morang, Nepal

**Keywords:** *atypical mycobacteria*, *non tuberculous Mycobacteria*, *rapidly growing Mycobacteria*

## Abstract

**Introduction:**

Non-tuberculous Mycobacteria are increasingly recognized, nowadays as an important pathogen in delayed surgical site infection in post operative cases. We here in describe cases of surgical site infection caused by Non-tuberculous Mycobacteria, seen in two centers in Jhapa. The aim of the study was to increase awareness of this atypical mycobacterial infection, prompt diagnosis, and treatment that may ultimately provide better care to patients.

**Methods:**

Forty four patients underwent different kinds of operations in two different private hospitals in Jhapa district of Nepal. All patients were presented with painful, draining subcutaneous nodules at the infection sites. Repeated aspiration of abscess, incision and drainage of the wound were done and specimen was sent for microbiological and histopathological examination. All patients were treated with repeated wound debridement and tab. Clarithromycin and inj. Tobramycin for 45days.

**Results:**

Mycobacterium Chelone were isolated from the purulent drainage obtained from wounds by routine microbiological techniques. Of the forty four cases, thirty of them had acid fast bacilli stain positive, two had acid fast bacilli culture positive. All the patients except two cases were treated with injection Tobramycin and Clarithromycin for six weeks.

**Conclusions:**

There should be high level of clinical suspicion for patients presenting with delayed post- operative wound infections for the diagnosis of non-tubercular mycobacreria as causative agents. These infections not only cause physical but also emotional distress that affects both the patients and the surgeon. Emphasis should be given on good sterilization technique to avoid such infections.

## INTRODUCTION

Non-Tuberculous Mycobacteria (NTM) are ubiquitous environmental acid-fast bacilli.^1^ NTM are rapid growing mycobacteria (RGM), it has been isolated from natural water, tap water, soil, etc.^2^ These atypical mycobacteria are resistant to sterilizers, antiseptics and standard disinfectants.^3^

RGM, the hydrophobic organism has the ability to form bio films for its successful survival in the environment. Shedding of these organisms from the bio film in a water pipe or device may be the reason for acquiring this infection.^4^ Diagnosis often delayed, as Ziehl Neeelsen and mycobacterial cultures are not routinely performed on skin biopsy specimens on surgical wound.^5^ Surgical site infections due to rapidly growing Mycobacteria cause not only physical disfigurement but also emotional distress to the patients.

Hence aim of our study was to increase awareness of this infection, prompt diagnosis, and treatment that may ultimately provide better care to patients.

## METHODS

A cross-sectional study was done from May 2014 to July 2015 at two different hospitals in Jhapa district of eastern Nepal. Target population were all post operative patients having surgical site infections. All the patients who developed surgical site infections after three weeks of operation were included in the study. Patients who developed surgical site infection in the immediate post operative period were excluded from our study. Total of 44 patients were included and data was collected by convenience sampling method.

Repeated aspiration of abscess, incision and drainage of the wound were done and specimen was sent for investigation. Microbiological investigation done was gram stain, pus for culture and sensitivity. Acid Fast Bacilli (AFB) stain and AFB culture on Lowenstein-Jensen (LJ) medium. All patients were treated with repeated wound debridement and tab. Clarithomycin and inj. Tobramycin for 45 days, Patients were followed up for 2 years after successful treatment. For data entry and analysis Microsoft Excel software was used.

## RESULTS

Among the 44 patients the majority of patients were females 33 (75%) females and 11 (25%) males with, median age of 28.4 years (range 20–47yrs).

All patients had undergone surgery in different surgical settings. The patient undergone open laparoscopic cholecystectomy (n = 15), caesarean section (n = 13), Herniotomy (n = 5), Appendicectomy (n=4), Transabdominal hysterectomy (TAH) (n=3), Hernioplasty (n=2), Excision biopsy (n=2) (Table 1).

**Table 1.** Types of surgery.

Surgery	n (%)
Appendectomy	4 (9)
Cholecystectomy	15 (34)
Heriniotomy	6 (13)
Caeserean Section	13 (29)
Excision Biopsy	2 (5)
Hernioplasty	1 (2)
TAH	3 (7)
TOTAL	44

No major disease comorbidities or cause of immuno suppression (e.g. HIV infection) were identified among the patients. Skin findings varied widely, non healing ulcers, subcutaneous abscesses, Subcutaneous fluctuant or firm nodules of varying size, and erythema including sinus tracts in association with ulcers or chronic drainage from prior surgical wounds. Systemic symptoms such as fever or a chill were as sent in all patients.

Ten patients were initially treated by repeated incision and drainage and were, started with antibiotics like cloxacillin, second-or third-generation Cephalosporin (such as Cefuroxime or Ceftriaxone), Metronidazole or Amoxillin/Clavulanate as culture were sterile. This treatment was repeated on several occasions ranging over periods of one month or more in some cases. The median time from surgical procedure to onset of infection was 27 days (range: 23 to 64 days) and the mean interval between clinical presentation and diagnosis was 39 days (range: 20–63 days).

Thirty samples revealed the presence of AFB on examination of smear prepared directly from specimens. Fourteen samples were AFB-negative. Two samples were sent to B.P. Koirala Institute of Health Sciences Dharan for culture and further analysis which confirmed M. chelonae and M. fortium. Two patients lost follow up.

As there is no well defined treatment protocol, patients managed according to the available literature from various sources. All patients initially underwent surgical procedures to drain existing abscesses and debridement. Of the 44 patients 42 patients received Clarithomycin and inj. Tobramycin for 45 days. Two patients lost follow up. One of the two patients with Hernioplasty, mesh was removed in one who presented late with fistula and abscess and one presented early, who got better only with antibiotic therapy. All the patients expect two who lost follow up responded well and were cured. Water tank for operation theatre supply and gluteraldehyde solutions and its chamber were changed. Laparoscopic hand instruments were sterilized by ethylene oxide method, meticulous cleaning of instruments was done and operation theatre was closed for seven days as preventive measure. No recurrence for two years of follow up and no more new cases identified.

## DISCUSSION

Atypical mycobacteria have been known to colonize tap water, natural waters, and soil and thus can easily contaminate solutions and disinfectants used in hospital settings. These infections have thus been a source of significant morbidity for patients recovering from open or laparoscopic surgeries.^9^

Other pyogenic bacteria have a short incubation period as compared to RGM which have a longer incubation period ranging from several days to several months.^10^ The absence of clinical response after the administration of antimicrobial agents against commonly invading bacteria (e.g., Staphylococci, Streptococci) and the sterility of routine cultures taken from the infected sites were clues for our infection.

The median time between the onset of symptoms and the micro-biological diagnosis was slightly more than three weeks. Therefore, a high index of suspicion is imperative for the diagnosis to be made. A study by Song et al.^10^ Stated that since the symptoms are relatively mild and indolent, the clinical diagnosis of Mycobacteriosis is often delayed and took more than two month from initial manifestation to diagnosis.

Delays of more than one year have been reported. A high degree of clinical suspicion and appropriate microbiological techniques are necessary to avoid delays in diagnosis delays in diagnosis.^11^ Indurations, abscess and discharging sinus are common local symptoms, but overt systemic illness is rarely found.^12^ The clinical features in our study were also similar, with erythematous nodules indurations, micro abscesses and discharging sinuses.

The presentation can be described in a few stages. The first stage is characterized by tender nodules with appear four weeks after the surgery and projects out in the vicinity of the surgery site. During the second stage, the nodules get bigger in size, become more tender and inflamed and eventually form a sinus discharging white pus. In the third stage of infection, there is a reduction of pain following discharge of pus with necroses of overlying skin. In the fourth stage, the area develops into a chronic sinus discharging white fluid followed by the fifth stage where the area darkens with necrosed skin. If left untreated, the infection can continue for months and multiple nodules appear in different areas. All our patients presented with only local manifestations that started with painful nodules which gradually increased in size, which then would fistulise and open on the skin draining pus while none of them had any systemic manifestations.

All our patients with post operative wound infections had repeated sterile aerobic and anaerobic cultures. Hence, it is very difficult to clinically diagnose RGM infections because they lack characteristic clinical features. Surgical site infections with abscess and chronic inflammation should be initially managed with conventional anti-biotic, however if here is no response and gram stain and routine culture are negative then Mycobacterial infection should be strongly suspected. Hence, AFB staining and culture for Mycobacterium should be done for sterile culture.

The source of infection in our country is not clear. In our current series the source of infection in patients who had undergone laparoscopy is primarily attributed to inadequate sterilization of laparoscope instruments. The possible reason is; the layer of insulation in the instrument restricts sterilization by auto- clave. Moreover, mechanical cleaning of blood and charred tissue that accumulates in the joints of the instruments are also not done properly after surgery. Hence, these contaminated instruments during the surgical process leave endospores on the subcutaneous tissue which grows and after an incubation period of 3–4wk clinical symptoms appear.^13^

Although many patients had underwent surgery in the same hospital and also under the same environmental conditions it is not clear why only few cases or only sporadic case that also had normal immune systems and no other predisposing medical condition have acquired the infection. Hence, future studies that focus on other conditions that predispose to infection are required.

There has been much controversy regarding the treatment for port-hole infections with atypical mycobacteria.

Once there is manifestation of clinical symptoms, the standard treatment consists of 28 day regimen of oral Clarithromycin and Ciprofloxacin or Amikacin. However, local administration of aminoglcyosides has been shown to be highly efficacious in the treatment of particularly stubborn nodules and sinuses that persist after completion of oral therapy.^14^

Successful treatment of RGM requires both surgical treatment and combination of antibiotics.^15^ Antibiotics should be given based on their susceptibility report and also combination of antibiotics is preferable over single regimen.^16^ M. fortuitum responds to antibiotics like Amikacin, quinolones, Doxycycline and Sulphamethaxole. A sperla test studies Clarithromycin, Cefoxitin and Imipenem were useful for treatment against RGM.^17–19^ Almost all the patients in our study were cured with a combined approach of drainage and Clarithromycin based combination therapy. In hernioplasty, patient mesh needs to be removed.

It has been recommended that to prevent recurrence, antibiotic treatment should be given for a minimum of at least three months, or at least 3 to 6 wk after the wound heals. Recent work has also recommended that antibiotics should be given for 6 to12 months though the optimal length of treatment has not been yet established.^20^

Current guidelines on infection control recommend a minimum exposure time of 8–12 hours to achieve the desired level of sporicidal activity of these germicides and the use of higher concentrations (3.4%) of glutaraldehyde disinfectants. Furthermore, proper disposal of glutaraldehyde based disinfectants is not followed. These chemical can be used for maximum of a 100 cycles or a period of 14 days (2.5% glutaraldehyde) or 28 days (3.4% glutaraldehyde), but often no count of cycles are kept in some hospitals and thus the chemicals often do not have the right potency to achieve the desired level of sterilization. Finally, the practice of rinsing the instruments with boiled tap water to rinse off the glutaraldehyde, further limits the efficacy of use of this system of sterilization as it causes the re-introduction of mycobacterial spores on the instruments that are then deposited at the ports.^21^ Secondly, it is necessary to limit glutaraldehyde disinfectants and replace it with ethylene oxide gas sterilization, as this has been shown to be highly effective in reducing atypical mycobacterial infections following laparoscopy.^22^

The use of advanced sterilization systems such as STERRAD, which uses gas plasma technology to kill spores at low temperatures, or the use of ethylene oxide gas is strongly recommended for sterilization of insulated laparoscopic instruments. Another option is to keep instruments for 24 hours in a formalin gas chamber. However, the instruments must be thoroughly cleansed and dried for this process to be effective, as the presence of dirt and moisture prevents the penetration of formalin gas, thus giving the same disastrous results. Finally, the use of disposable laparoscopic instruments, as is done in Western countries, is strongly advocated.

The limitation of this study could be that conventional method was utilized in the study. Molecular diagnostics like Polymerase Chain Reactions and culture and antibiotic sensitivity couldn't be done in all cases due to financial constraints. Source of infection could not be identified. Also, convenient sampling method utilized has biasness.

## CONCLUSIONS

Though rare, mycobacteria should be considered as differential diagnosis of late onset Surgical site infection (SSI). Strict sterilization procedure should be followed up in hospital. Ethylene oxide plant and autoclaving are more effective methods, than gluteraldehyde. Finally, the use of disposable laparoscopic instruments, as is done in developed countries, is strongly advocated.
